# Separation and Lipid Inhibition Effects of a Novel Decapeptide from *Chlorella pyenoidose*

**DOI:** 10.3390/molecules24193527

**Published:** 2019-09-29

**Authors:** Ruilin Zhang, Jian Chen, Xinwu Mao, Ping Qi, Xuewu Zhang

**Affiliations:** 1 College of Food Science and Engineering, South China University of Technology, Guangzhou 510640, China; ruilin_zhang92@163.com (R.Z.); fejchen@scut.edu.cn (J.C.); 2 Era (China) Company Ltd., Shenzhen 518115, China; 3 Guangzhou Institute for Food Inspection, Guangzhou 510640, China; gzsp2000@163.com

**Keywords:** *Chlorella pyrenoidosa* peptide, lipase inhibitor, 3T3-L1, AMPK pathway

## Abstract

A novel lipid inhibition peptide Leu-Leu-Val-Val-Try-Pro-Trp-Thr-Gln-Arg (PP1) (MW 1274.53 Da) was obtained from *Chlorella pyenoidose* using enzymatic hydrolysis, gel filtration chromatography, and LC–MS/MS. Its lipid inhibition effects indicated that the synthetic peptide PP1 exhibits a good inhibitory effect against porcine pancreatic lipase (PL) (47.95%) at 200 μg/mL, which could be attributed to its hydrogen binding into catalytic sites of PL (Ser153, Asp177, and His 264) by docking analysis. Furthermore, in 3T3-L1 cells, the synthetic PP1 remarkedly decreased the accumulation of intracellular triacylglycerol (27.9%, 600 μg/mL), which carried a similar consequence as the positive drug simvastatin (24.1%, 10 μM). Western blot revealed that PP1 inhibited the lipid accumulation and fatty acid synthesis in 3T3-L1 adipocytes in two pathways, primarily: nonalcoholic fatty liver disease (NAFLD) pathway (C/EBPα, SREBP-1c, AMPKα) and AMPK signaling pathway (SREBP-1c, PPARγ, AMPKα). In short, these results support that PP1 can be used as a potential agent against obesity.

## 1. Introduction

Bioactive peptides are generally short peptides (2–20 amino acids) that are partially obtained by enzymatic hydrolysis from protein and are important parts in food-science studies. Many different parameters affecting the bioactivity are explored, such as the source of protein, the degree of hydrolysis, peptide structure, amino acid composition, molecular weight (MW), and type of protease [1]. Bioactive peptides from various sources have certain functions, such as antitumor effects [2,3], antihypertensive effects [4], antioxidant activity [5,6], etc. *Chlorella*, a type of single-cell green algae, contains over 50% bioactive proteins and has become a major source for extracting high-yield proteins. It is reported that *Chlorella* has certain functional properties, such as antioxidant [7], anti-inflammatory [8], antihypertensive [9], antitumor activities [2], etc.

Obesity is essentially an excessive energy intake that is stored in the form of triacylglycerols in fatty tissue. Excessive accumulation of triacylglycerols in fatty tissue causes a disorder of lipid metabolism. Presently, obesity is a prevalent disease and has become a major problem in today’s society. Lipases, a subclass of the esterases, catalyze the hydrolysis of fats and oils. For humans, pancreatic lipase is a key lipase for lipid absorption throughout the body and is responsible for the hydrolysis of total dietary fats [10]. Pancreatic lipase serves to transport dietary lipids (e.g., triglycerides and oils) into simple molecules through lipid emulsification that is easily absorbed in the intestine. Therefore, inhibition of pancreatic lipase decreases the absorption of lipids, which can reduce obesity [11,12,13].

Previous studies have reported that preventing differentiation of preadipocytes to adipocytes and inhibiting lipid accumulation contribute to anti-obesity [14]. The process of adipogenesis also involves changes in the expression of adipogenic-specific proteins. In mammalian cells, the peroxisome proliferator-activated receptor γ (PPARγ), CCAAT/enhancer binding protein α (C/EBPα), and sterol regulatory element-binding protein 1c (SREBP-1c) [15] are known to act as important regulators of adipogenesis. PPARγ is essential for differentiation of adipocyte as an inducer during differentiation of preadipocytes to adipocytes. Without it, precursor cells are unable to differentiate into mature adipocytes. Additionally, adenosine monophosphate-activated protein kinase (AMPK) is an important mediator involved in regulating energy balance in the human body [16], and activating AMPK could be a therapy for inhibiting fat accumulation in adipocytes. 

Many recent studies have focused on developing anti-obesity agents from natural substances. However, few reports are available on the lipid inhibition effects of peptides from seaweeds. This research focused on purification and identification of bioactive peptide fractions from enzymatic hydrolysis of *C. pyenoidose*. Furthermore, its effect on the inhibition of the pancreatic lipase (PL) activity and suppression of fat accumulation in 3T3-L1 cells were investigated.

## 2. Results

### 2.1. Characterization of the Extracted Proteins and Enzymatic Hydrolysates

In this study, about 72.4% of whole proteins were extracted from *C. pyenoidose* by using a series of steps [17]. To obtain potential PL inhibitory peptides from *C. pyenoidose*, the extracted proteins were enzymatically hydrolyzed with four proteases: pepsin, papain, trypsin, and Alcalase. For the extracted proteins, the percentages of molecular weight (MW) were 40.37% (<3000 Da), 11.02% (<1000 Da), and 29.29% (1000~3000 Da) (Table 1), which were similar with those in pepsin hydrolysate. For Alcalase hydrolysate, the percentage of MW in the range <3000 Da was highest (51.17%) among all the enzymatic hydrolysates. 

### 2.2. PL inhibitory Activities of Proteins and Enzymatic Hydrolysates

**Figure 1:**
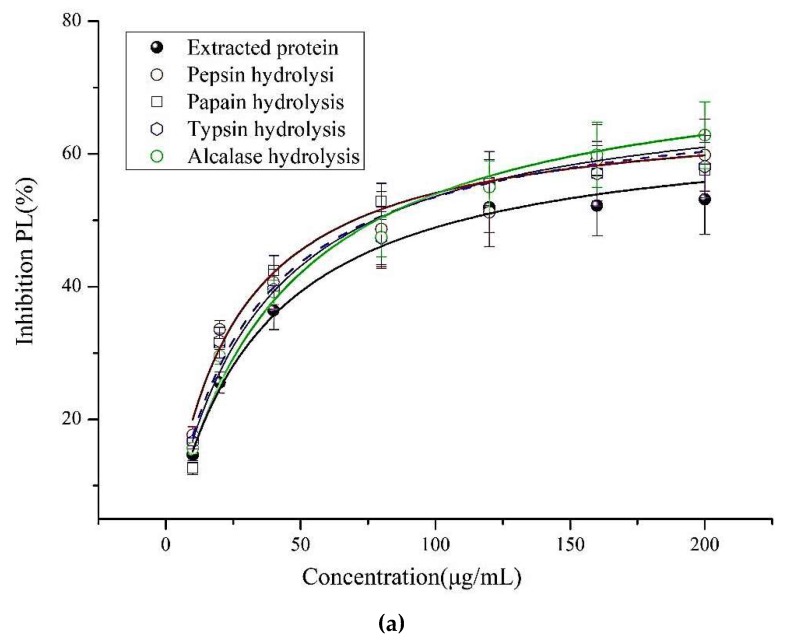

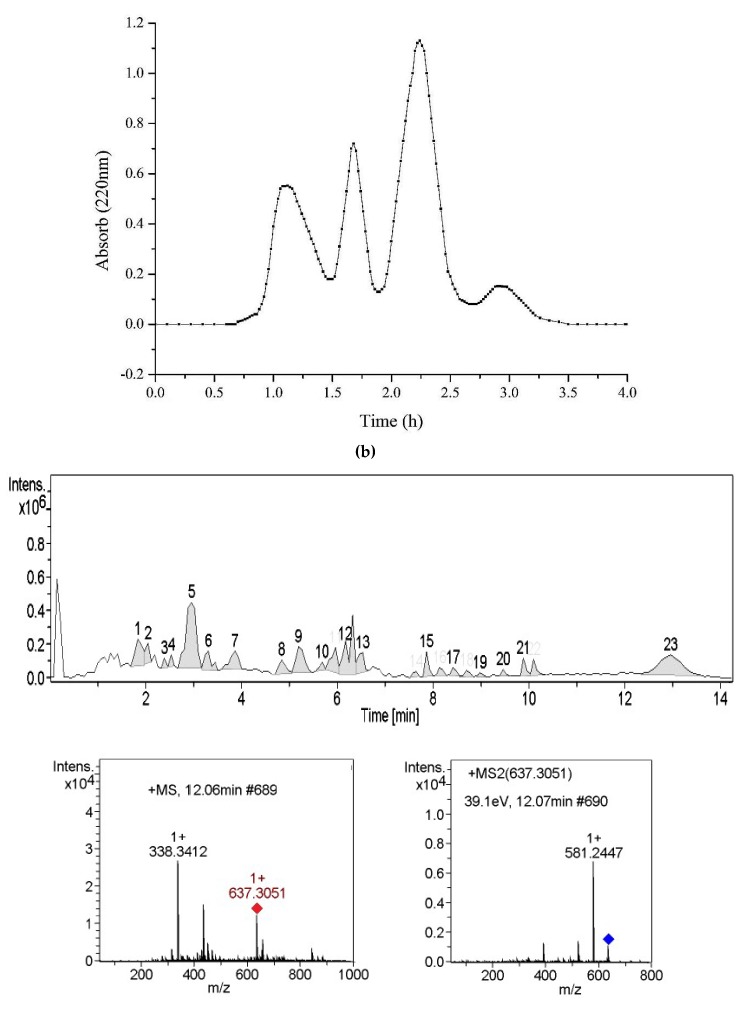

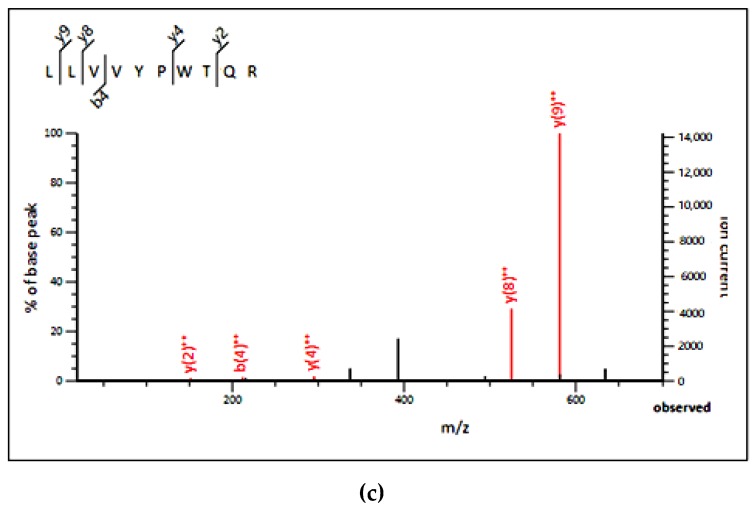
(**a**) The curve of pancreatic lipase inhibitory activity of extracted proteins and enzymatic hydrolysates by Copper soap method; (**b**) elution curve of Alcalase hydrolysis on G25 (<5K); (**c**) LC–MS chromatogram of the active fraction A2 from RP-HPLC; MS, MS/MS spectra, and y and b ions spectra of PP1.

To clarify the inhibitory effects on PL, various concentrations of proteins and hydrolysates against PL activities were examined in vitro. In Figure 1a, Alcalase enzymatic hydrolysate exhibited the greatest PL inhibitory activities among the enzymatic hydrolysates with IC_50_ 87.79 ± 5.12 μg/mL, followed by the pepsin hydrolysate, which had an inhibition on PL activity that was IC_50_ 89.65 ± 7.06 μg/mL. In Copper soap assay, the order of PL inhibitory capacities is Alcalase hydrolysate > pepsin hydrolysate > trypsin hydrolysate > papain hydrolysate > protein. 

### 2.3. Separation of Alcalase Hydrolysates and PL Inhibition 

Subsequently, Alcalase enzymatic hydrolysate was fractioned by ultrafiltration membrane (<5 and >5 kDa). The PL inhibitory capacities of different fractions were determined (Table 2). Results indicated that the hydrolysate (<5 kDa) exhibits a better inhibition on PL than another one. Thus, a molecular weight (<5 kDa) of Alcalase enzymatic hydrolysate was selected for further purification using gel chromatography Sephadex^®^ G-25, and four fractions (A1–A4) were obtained (Figure 1b). Data in Table 2 show that the A2 exhibits the strongest inhibition among fractions by the Copper soap method.

### 2.4. Identification of PL Inhibitory Peptides

In order to identify peptides, A2 was subjected to LC–MS/MS analysis. The results of LC–MS/MS chromatogram are shown in Figure 1c. After annotation by Mascot sequence matching software (Matrix Science) with Ludwig NR Database, six peptide sequences (SISISVAGGGR, LLVVYPWTQR, SDDPHTFGQGTK, SRQLTLYPGAER, KNGAPAEK, and KQTALVELVK) were identified from A2 fraction (Table 3), which were originated from fructose-bisphosphate aldolase 1, Ribosomal protein S of Chloroplastic, Protein brassinosteroid Transmembrane protein, ADP-ribosylation factor, GTPase-activating protein and Ribosomal protein S2, Chloroplastic in *C. pyenoidose*, respectively. Based on top three peptides (SISISVAGGGR, LLVVYPWTQR, and SDDPHTFGQGTK) with high bioactivity prediction score (0.45, 0.38, and 0.35, respectively) by Peptide Ranker (http://bioware.ucd.ie), a short peptide LLVVYPWTQR (PP1) was selected for further study, which can also properly bind to PL surface using Pep-site (http://pepsite2.russelllab.org/). According to MS profile (Figure 1c), the content of PP1 was estimated as 1.93% by calculating area. Then the decapeptide was synthesized, and its purity and molecular weight were determined by HPLC–MS analysis: 97.06% and 1274.53 Da, respectively. 

### 2.5. PL Inhibitory Activities of the Synthesized Peptide 

**Figure 2:**
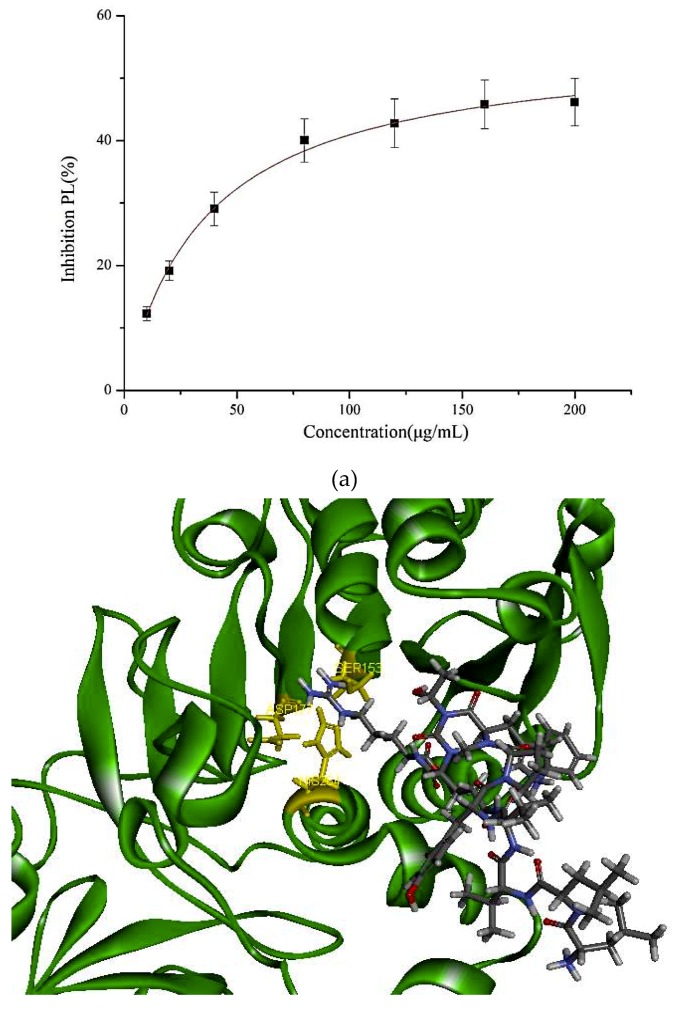

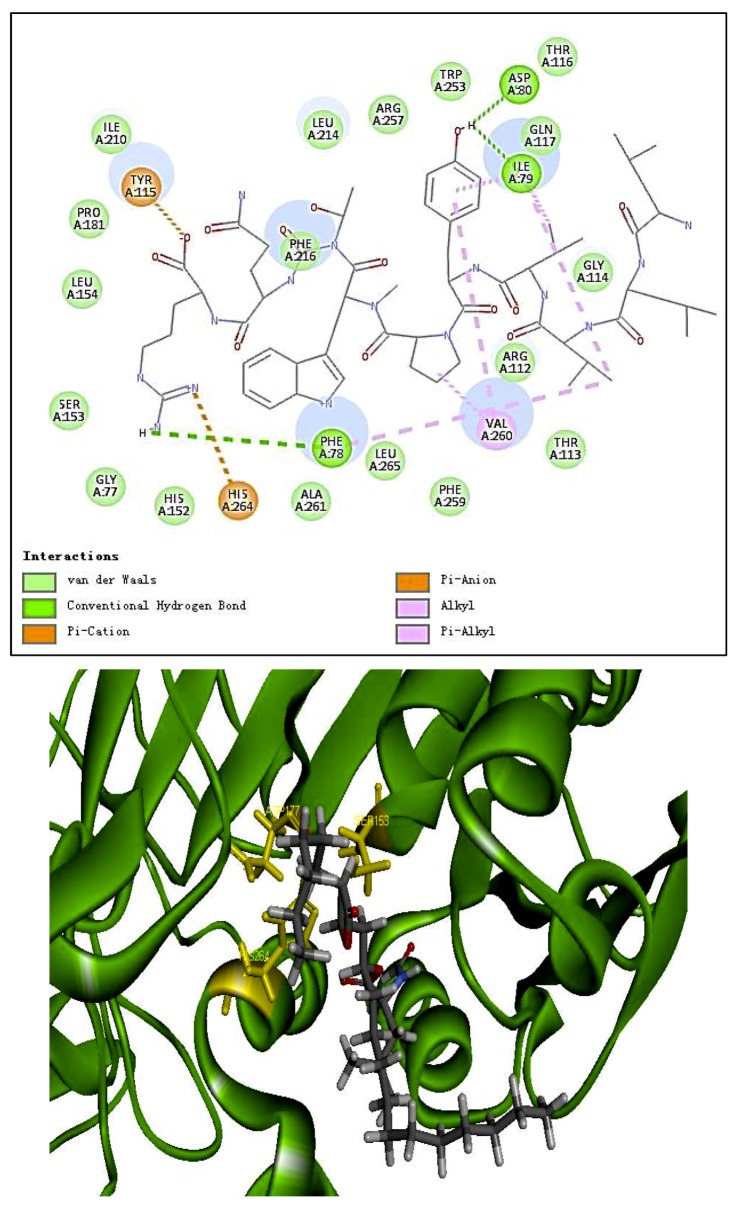

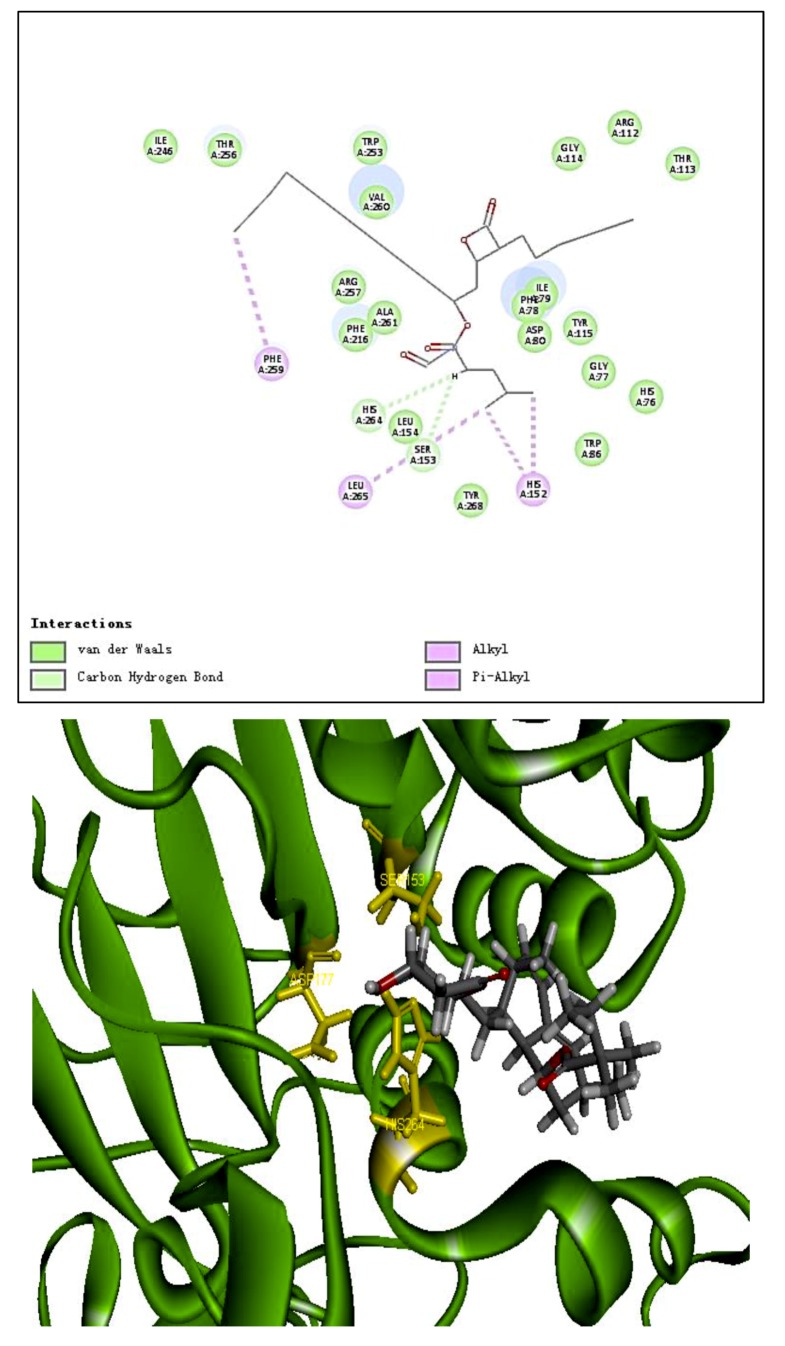

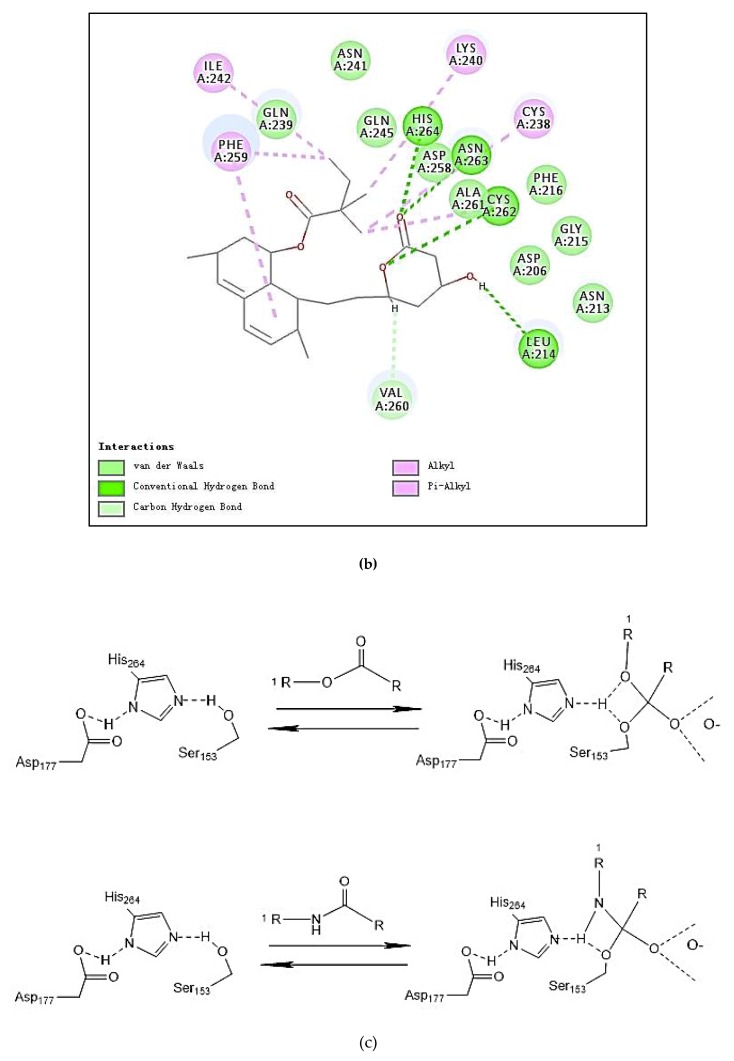
(**a**) Inhibition of lipase by the synthesized peptide by Copper soap method. (**b**) Docking analysis of the porcine pancreatic lipase (PDB code: 1ETH) with the decapeptide, showing hydrogen bond (blue sagittate), Van der Waals (green), and Electrostatic (pink). Two-dimensional diagrams of predicted interactions between the decapeptide and the porcine pancreatic lipase (PDB code: 1ETH), (A) PP1 (B) simvastatin, and (C) Orlistat. (**c**) Catalytic mechanism of lipase with lipid and polypeptide.

In our study, the PL inhibitory effect of the synthetic PP1 is shown in Figure 2. The data show that PP1 exhibited a good inhibitory effect on PL (47.95% ± 2.3%) at 200 μg/mL. 

### 2.6. Docking Studies 

The molecular structure of PP1 was built by Chem 3D Pro 14.0. The PP1 was docked into the rigid active site of PL using Libdock Discovery Studio 3.5 Software. In order to understand the binding modes of PP1 bound into PL, molecular docking of the ligands PP1, simvastatin, and Orlistat with the receptor PL (PDB 1ETH) were performed to analyze the hydrogen bond, electrostatic interactions, and hydrophobic interactions found between the ligands and the active site of PL. The conformations revealed the types of intermolecular interactions between the ligand and the PL pocket, as shown in Figure 2b. Molecular docking of inhibitors into the active site of the PL resulted in various poses with different scores (Table 4). In Table 4, the specific interactions indicated that the absolute values for the steric and the hydrogen bonds energy of PP1 are low; the effective docking of PP1 into the active site of PL revealed that the porcine lipase inhibitory activity of PP1 is involved in its binding to catalytic sites of the lipase (Ser153, Asp177, and His 264). 

### 2.7. Effects of the Decapeptide PP1 on Lipid Accumulation in 3T3-L1 Cells

**Figure 3:**
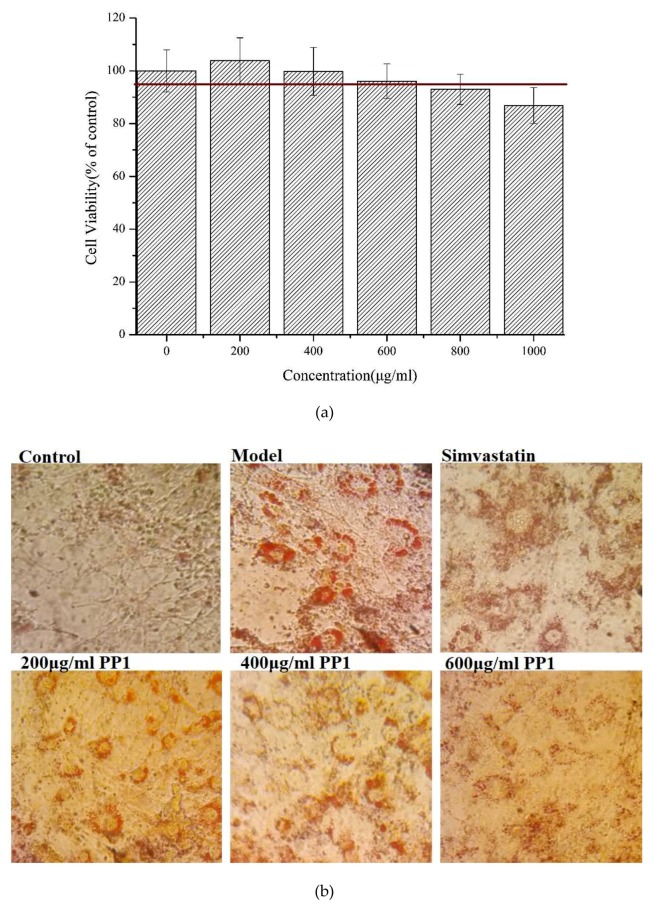

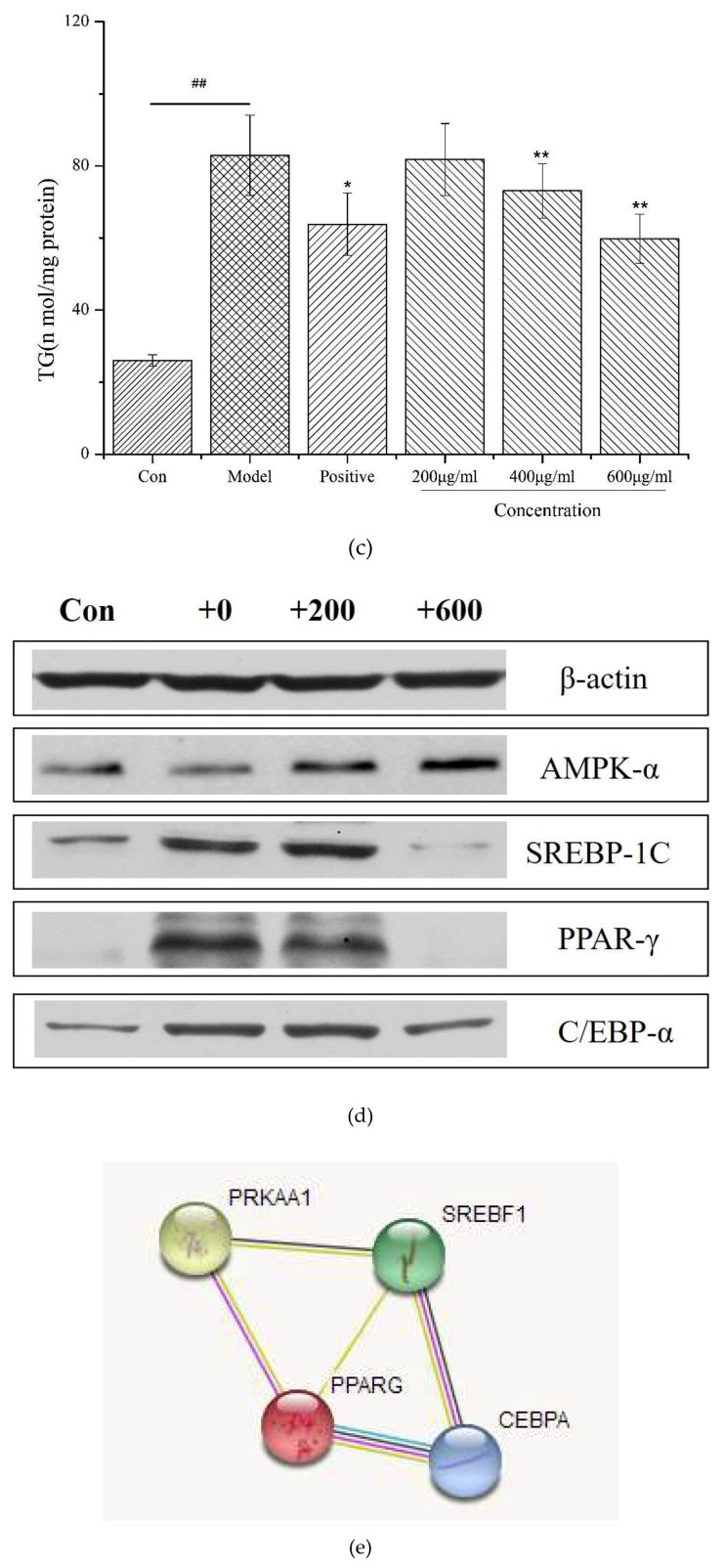
(**a**) The effect of PP1 on 3T3-L1 cell viability. (**b**) Lipid accumulation was determined by Oil Red O. (**c**) PP1 inhibited intracellular lipid accumulation in 3T3-L1 adipocytes, 10 μM simvastatin was used as a positive group. (**d**) The protein expressions by Western blot. (**e**) The core network mapped by STRING analysis, CEBPA–C/EBPα, SREBF1–SREBP-1c, PPARG–PPARγ, PRKAA1–AMPKα.

Figure 3a shows that the synthetic decapeptide PP1 should not affect cell viability at 0–600 μg/mL. In a further study, we investigated the lipid regulation of PP1 in adipocytes. The 3T3-L1 cells were used to differentiate into adipocytes under various concentrations of PP1, and thereafter the intracellular lipids were stained with Oil Red O. The number of intracellular lipid droplets was clearly decreased compared with that in the model group (Figure 3b). Then, the levels of intracellular triacylglycerol in PP1-treated cells were measured. The intracellular triacylglycerol in the mature adipocyte (82.98 ± 11.2 nmol/mg protein) was significantly enhanced compared with that in the pre-adipocytes (25.98 ± 1.6 nmol/mg protein). In contrast, PP1 significantly reduced the accumulation of intracellular triacylglycerol (59.82 ± 6.78 nmol/mg protein, *p* < 0.05) at 600 μg/mL (Figure 3c). These results indicate that PP1 has a good suppressive effect on the lipid accumulation in 3T3-L1 cells.

### 2.8. Effects of the Decapeptide PP1 on the Expression of Protein Levels in 3T3-L1 Cells

To investigate the molecular mechanisms underlying the inhibitory effect of PP1 on 3T3-L1 adipocyte differentiation, we measured the expression of some adipogenesis-related proteins in adipocyte cells. As shown in Figure 3d, the levels of the adipogenic-specific proteins C/EBPα, SREBP-1c, and PPARγ significantly increased in the model group, compared to the pre-adipocytes. PP1 treatment (600 μg/mL) significantly increased the expression of AMPKα and decreased the levels of C/EBPα, SREBP-1c, and PPARγ signaling in 3T3-L1 adipocytes. Collectively, these results indicate that PP1 could inhibit fat accumulation and fatty acid synthesis in adipocyte cells by activating the AMPK pathway and down-regulating adipogenic-specific proteins, including C/EBPα, SREBP-1c, and PPARγ, in 3T3-L1 cells.

## 3. Discussion

In the literature, PL is a key enzyme in fat digestion, and its inhibition is associated with decreased fat absorption. Previous reports have shown that lipase inhibitors can effectively reduce fat absorption to suppress weight gain in obese patients [13,18]. Computer-based virtual simulation is possible for inhibition mechanism analysis of peptide on PL. A virtual methodology was used for the assessment of the inhibitory potential. The inhibition mechanism on pancreatic lipase of the decapeptide PP1 was demonstrated by the tool Discovery Studio (3.5) Libdock, with Orlistat and simvastatin as the contrasts. Ligands (PP1, Orlistat, and simvastatin) were acting inhibitors of pancreatic lipases by forming some covalent bonds with the active serine site of lipase and inactivating them to hydrolyze dietary fat. Galloyl and hydroxyl groups in peptide drugs and controls could contribute to the ligand–lipase binding, due to hydrophobic interactions of peptides with the interior hydrophobic groups of lipases and the hydrogen bonds between the Galloyl and hydroxyl groups. The proposed catalytic mechanisms of lipase with lipid and polypeptide were described in Figure 2c. 

Obesity is regarded as a complex process of glucose uptake, lipid synthesis, and fat accumulation in adipocytes, involving different metabolic signaling pathways [15]. Evidences showed obesity is influenced by numerous factors, such as the energy balance, biological factors, and hormones/cytokines/other factors [15,16]. However, few reports are available on the lipid inhibition effects of bioactive peptides from *C. pyenoidose*. In the present study, investigations were done to evaluate the lipid inhibition effects of PP1 and its mode of action in adipocytes. Adipocytes play a vital role in the regulation of energy intake, energy expenditure, and both lipid and carbohydrate metabolism; thus, they lower lipogenesis, enhance lipolysis, and decrease pre-adipocyte differentiation, all of which are strategies against obesity [14,15,19]. The adipocyte-specific proteins, including C/EBPα, SREBP-1c, and PPARγ play essential roles during the differentiation of preadipocytes into adipocytes. In this study, PP1 displayed good differentiation inhibition activity. The present data show that the expression of C/EBPα, SREBP-1c, and PPARγ decreased significantly at a high concentration of PP1, compared to the model, which means that a high dose of PP1 could prevent pre-adipocyte 3T3-L1 differentiation. Moreover, recent reports suggested that AMPK is a major regulator of whole-body energy homeostasis, which gets activated by lower intracellular ATP levels [20]. In the present experiment, PP1 treatment could significantly increase AMPK-α signaling, and the activated AMPK may inhibit overall energy synthesis and enhance energy metabolism to inhibit fat accumulation in adipocytes. In addition, PP1 down-regulated the expressions of C/EBPα, SREBP-1c, and PPARγ, hence exerting influences on the differentiation of preadipocytes into adipocytes. STRING analysis revealed that the altered proteins (AMPK-α, C/EBPα, SREBP-1c, and PPARγ) are mapped into a core network (Figure 3e), which was primarily associated with two pathways: nonalcoholic fatty liver disease (NAFLD) pathway (C/EBPα, SREBP-1c, AMPKα) and AMPK signaling pathway (SREBP-1c, PPARγ, AMPKα).

It was assumed that the interaction of ligand-enzyme would be in charge of inhibiting the enzyme activity. The conformational changes of the protein and peptide structure can influence the catalytic activity of enzymes [21]. The structure–activity relationship between PL and its inhibitory peptides, as well as the inhibitory effects of PL in vivo, will undoubtedly be the next work in the future. 

## 4. Materials and Methods

### 4.1. Materials and Chemicals

*C. pyrenoidosa* powder (62.4% of total protein contents) was provided by Professor Zhang Daojing, East China University of Technology, Shanghai, China. BCA Protein Assay Kit and Triglyceride Assay Kit were purchased from Nanjing Jiancheng Bioengineering Institute (Nanjing, China). Pepsin (300,000 U/g), papain (800,000 U/g), and trypsin (1:250 U/g) were obtained from Guangzhou Qiyun Biotech Co., Ltd., Guangzhou, China. Alcalase 2.4 L (P4860), 3-(4,5-dimethylthiazol-2-yl)-2,5-diphenyltetrazolium bromide (MTT), pancreatic lipase, Orlistat, and simvastatin were obtained from Sigma, San Jose, CA, USA. Primary and secondary antibodies were purchased from Cell Signaling Technology (Danvers, MA, USA). Gel filtration chromatography Sephadex G-25 was from Whatman Liluo Science & Technology Instrument Co., Guangzhou, China. Other reagents were of analytical grade and were commercially available.

### 4.2. Hydrolysis of Proteins 

The proteins were extracted from *C. pyrenoidosa* according to how they were extracted in previous studies [17]. Two grams of proteins were dissolved in 10 mL water (w/v = 1:5) and separately hydrolyzed with four proteases (pepsin, papain, trypsin, and Alcalase) under controlled conditions for 5 h. Specifically, for pepsin, the ratio of enzyme to substrate (E/S) 6% (w/w), pH 3, and temperature 50 °C; for papain, the ratio of enzyme to substrate (E/S) 3% (w/w), pH 7, and temperature 50 °C; for pepsin, pH 2, temperature 37 °C, and the ratio of enzyme to substrate (E/S) 6% (w/w); for trypsin, p the ratio of enzyme to substrate (E/S) 3% w/w, pH 8, and temperature 50 °C; for Alcalase, the ratio of enzyme to substrate (E/S) 3% (w/w), pH 8, and temperature 50 °C. After hydrolysis, enzymes were inactivated by placing the samples in boiling water for 10 min. The hydrolysates were then centrifugated at 8000 r/min for 10 min. The supernatants were lyophilized and stored at −20 °C.

### 4.3. Molecular Weight Distributions 

Molecular weight distributions of extracted protein and enzymatic hydrolysates were determined by HPLC. The gel column was TSK gel G2000 SWXL, and the eluate was monitored under λ = 220 nm. The flow phase was composed of phosphate buffer (pH = 7.0) and the velocity of flow was 0.5 mL/min. 

### 4.4. Separation by Ultrafiltration

The enzymatic hydrolysates supernatants were subjected to ultrafiltration through molecular weight cut-off (MWCO) membrane of 5 kDa (Millipore Co., San Diego, CA, USA). Each fraction (<5 and >5 kDa) was collected, and its PL inhibitory activity was measured. The fractions were lyophilized and stored at −20 °C. 

### 4.5. Gel Filtration Chromatography 

The highest active fraction was separated by gel filtration chromatography. Two milligrams of bioactive fractions dissolved in distilled water at a concentration of 40 mg/mL were loaded onto a Sephadex G-25 (1.5 × 60 cm^2^). The column was eluted with distilled water at a flow rate of 0.5 mL/min. The eluate was detected at 280 nm. The eluates at the same peak were collected and then freeze-dried and stored at −20 °C. The PL inhibitory activity of each fraction was measured. 

### 4.6. Analysis by LC–MS/MS

The sequence of peptides of the collected peak with the highest lipase inhibition was identified by LC–MS/MS (maXis impact, Bruker, Germany). Specifically, the sample was diluted 1:5 with eluent A (0.1% formic acid in Milli-Q water) and injected into a C18 column (Waters Acquity UPLC BEH 300; 2.1 mm × 100 mm, 1.7 μm) with a flow rate of 0.3 mL/min and a column temperature of 30 °C. The peptides were separated using the following gradient: 6 ultraperformance liquid chromatography (UPLC) for peptide separation in a C18 column (2.1 × 120 mm) (Waters Corporation, Milford, MA, USA). Ten microliters of each peptide sample (0.4 mg/mL) were loaded for each elution. The elution was made in a linear gradient mode from 0% acetonitrile to 40% acetonitrile (20 min) at a flow rate of 0.3 mL/min. Data of MS/MS were recorded using mascot generic format (MGF) files, which were submitted to Mascot search engine V 2.3 (Matrix Science, UK), and sequences of peptides were identified based on a search in the database (Ludwig NR Database). The data acquisition parameters were as follows: electrospray ionization (ESI) source type, positive ion mode, scan region 50–1500 m/z, capillary 3500 V, charging voltage 2000 V, corona 0 nA, nebulizer 0.3 bar, dry heater 180 °C, APCI heater 0 °C, and dry gas 4.0 L/min.

### 4.7. Peptide Synthesis

Based on the bioactivity prediction by Peptide Ranker (http://bioware.ucd.ie) and Pep-site (http://pepsite2.russelllab.org/), the biological peptide LLVVYPWTQR (PP1) was chemically synthesized by GenScript Corporation (Shang Hai, China). The purity of the synthesized PP1 was verified to be higher than 95% by analytical HPLC coupled with MALDI–TOF MS.

### 4.8. Lipase Activity Assay

Copper soap method [22]: The lipase activity assay involved a reaction medium (10 mL) containing 1 mg of olive oil, 3 mL 0.0667 mol/L, pH = 7.38 phosphate buffer saline (PBS), and 0.1 mL (C = 1 mg/mL) of lipase in the presence of 1 mL peptides with different concentrations. The peptides solution was prepared by dissolving in the working solution and was then added to reaction mixtures just before the reaction. Control lipase activity was determined without peptides. All samples were analyzed in triplicate.

(1) $$\mathrm{Inhibition}\%=1-\frac{\left(Aa-A0)-(Ab-A0\right)}{\left(Aa-A0\right)}\times 100\%$$

where *Ab* refers to the absorbance value of the normal group; *A*0 is referred to the absorbance value of blank; and *Aa* is the absorbance values of the control group. 

### 4.9. Docking

The 3-D coordinates of the protein complex with the PL (PDB code: 1ETH) were downloaded from the Protein Data Bank(http://www.rcsb.org). The peptide (PP1) was modeled by using Libdock. Orlistat and simvastatin were used as positive controls. The Discovery studio software was used for the docking of PP1, simvastatin, and Orlistat to the PL protein. The potential binding sites (also referred to as cavities or active sites) were being identified using the built-in cavity detection algorithm, expanded Van der Waal with 9 Å Probe size. The docking modes were obtained with scoring results about the ligand–receptor combination, which could be used for PL inhibitory activity prediction. Finally, the high score of the poses correctly bound to the active site was evaluated. 

### 4.10. Lipid Inhibition Assay in Cells

First, 3T3-L1 cells were cultured in Dulbecco’s modified Eagle’s medium (DMEM) medium containing 10% (v/v) fatal bovine serum (FBS) and 1% antibiotics in a humidified atmosphere of 5% CO_2_ at 37 °C. For cell toxicity assessment, 3T3-L1 cells (1 × 10^5^) were seeded in a 96-well plate for 24 h. After that, the cells were incubated for 6 days in DMEM containing various concentrations (0–1.0 mg/mL) of the synthetic PP1. Cell toxicity was measured by MTT according to the instructions indicated by the manufacturer. For cell differentiation, 3T3-L1 cells were seeded in 6-well plates at a density of 2 × 10^5^ cells/well. Then, the medium was changed by DMEM with insulin (5 μg/mL; Sigma), 1 μM of dexamethasone (Sigma), and 0.5 mM of 3-isobutyl-1-methylxanthine. On day 3, the medium was replaced with DMEM containing insulin (5 μg/mL; Sigma) and 10% (v/v) FBS every 3 days. Various concentrations of the synthetic PP1 were attendant during the whole process of differentiation induction. Oil Red O staining of the intracellular lipids was carried out, as described previously. Moreover, intracellular triacylglycerol levels were measured by using an Assay Triglyceride Kit. Protein concentrations were determined with BCA Protein Assay.

### 4.11. Western Blot Analysis

Cells were lysed in Radio Immunoprecipitation Assay (RIPA) buffer for 1 h and then centrifuged at 12,000 rpm for 25 min at 4 °C. The protein concentrations were determined by using a BCA protein assay kit. The supernatants were mixed with 4 × Sodium dodecyl sulfate (SDS) sample buffer and boiled for 10 min. The samples were run on a 4–15% SDS polyacrylamide gel and then transferred to a polyvinylidene difluoride membrane. The membrane was blocked with 5% (w/v) bovine serum albumin (BSA) for 30 min and then incubated with primary antibodies at 4 °C overnight. After washing with Tris-Buffered Saline and Tween 20 (TBST) 3 times, the membranes were washed with TBST and incubated with the secondary antibodies. Signals were detected with a scanner (Thermo Fisher Scientific, Inc.), and the fluorescence intensity was measured using Image software. 

### 4.12. Statistical Analysis

Data were expressed as mean ± standard deviation of the number of replicates performed in the experiment. The significance level (*p* < 0.05) was determined by a paired t-test. All statistical analyses were performed using SPSS (9.0).

## 5. Conclusions

In conclusion, the present data indicate that the decapeptide PP1 (Leu-Leu-Val-Val-Try-Pro-Trp-Thr-Gln-Arg) possesses inhibitory activity on pancreatic lipase. PP1 can inhibit fatty acid synthesis and fat accumulation in the adipocyte. This suggests the potential of PP1 as a therapeutic agent against obesity.

## Figures and Tables

**Table 1:** Molecular weight distributions.

MW (Da)	Percentage of Molecular Weight (%)				
	Original Protein	Pepsin Hydrolysate	Papain Hydrolysate	Trypsin Hydrolysate	Alcalase Hydrolysate
>10000	13.95	7.14	5.98	5.26	4.83
10000~5000	18.95	16.50	12.95	11. 45	9.95
5000~3000	27.38	36.34	31.87	33.37	32.51
3000~1000	29.29	33.02	35.65	35.82	38.63
<1000	11.03	9.03	13.27	14.06	13.81

**Table 2:** Inhibition of pancreatic lipase activity of Alcalase hydrolysate fractions.

Groups	Inhibition of Pancreatic Lipase (%)
>5 kDa	41.4 ± 2.75
<5 kDa	75.73 ± 2.88
A1	54.51 ± 1.93
A2	74.05 ± 3.7
A3	60.5 ± 1.89
A4	63.5 ± 2.81
Positive drug: simvastatin	35.2 ± 1.9
Positive drug: Orlistat	88.85 ± 2.42

**Table 3:** Results of the identified peptides by Mascot Science and their bioactivity prediction by Peptide Ranker.

NO. Peptide	Identified Peptide SEQUENCE	Observed	Mr(expt)	Mr(calc)	Protein in *C. pyenoidose*	Bioactivity Prediction Score
1	SISISVAGGGR	531.28	1060.54	1059.56	Fructose-bisphosphate aldolase 1	0.45
2	LLVVYPWTQR	637.31	1272.59	1273.71	Ribosomal protein S, Chloroplastic	0.38
3	SDDPHTFGQGTK	645.80	1289.61	1288.56	Protein brassinosteroid insensitive	0.35
4	SRQLTLYPGAER	695.46	1388.89	1388.73	Transmembrane protein	0.17
5	KNGAPAEK	408.23	814.44	813.43	ADP-ribosylation factor GTPase-activating protein	0.15
6	KQTALVELVK	377.18	1128.52	1127.69	Ribosomal protein S2, Chloroplastic	0.09

**Table 4:** Detailed view of the docked pose of the corresponding interacting amino acids within the binding site of pancreatic lipase enzyme (PDB code:1ETH).

Descriptor	LLVVYPWTQR	Orlistat	Simvastatin
Docking sore	147.67	152.887	124.76
Residues formed hydrogen bonds with the ligand	Ile79, Asp80, Tyr115, Ser153	_	Leu214, Cys262, Asn263, His264
Pi interaction	0	4	1
Number of amino acids	20	24	15
Number of hydrogen bonds	6	0	4
